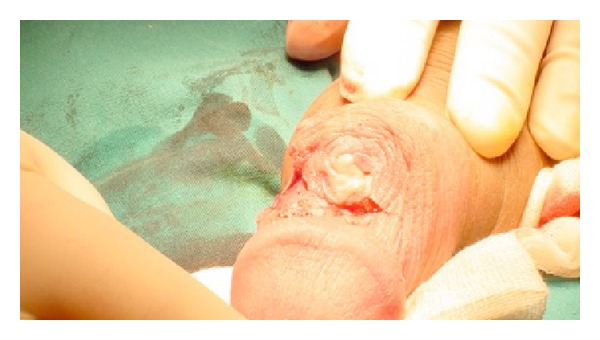# Pilonidal Cyst of the Penis Mimicking Carcinoma

**DOI:** 10.1155/2013/984757

**Published:** 2013-09-17

**Authors:** Luigi Cormio, Francesca Sanguedolce, Paolo Massenio, Giuseppe Di Fino, Giuseppe Carrieri

**Affiliations:** ^1^Department of Urology and Renal Transplantation, University of Foggia, Viale Luigi Pinto 1, 71121 Foggia, Italy; ^2^Department of Pathology, University of Foggia, Viale Luigi Pinto 1, 71121 Foggia, Italy

## Abstract

Pilonidal sinus is a long-standing chronic inflammatory condition consisting of a sinus tract from the skin-lined orifice extending into subcutaneous tissue, with hairs attached to the wall of the tract and projecting outside of the opening. Penile location is rare, and differential diagnosis with severe balanoposthitis, epidermal cysts, and neoplasms can be difficult. We report a rare case of pilonidal cyst located between coronal sulcus and prepuce which, due to its ulcerated aspect and absence of a tract with projecting hairs, simulated a penile carcinoma.

## 1. Introduction

Pilonidal sinus is a long-standing chronic inflammatory condition consisting of a sinus tract from the skin-lined orifice extending into subcutaneous tissue, with hairs attached to the wall of the tract and projecting outside of the opening. It occurs most commonly in the sacrococcygeal area but can be seen also in scalp, ear, brow, cervical subcutaneous region, axilla, interdigital clefts, anterior chest wall, nipple, umbilicus, suprapubic region, perineum, clitoris, anal canal, sole of foot, and amputation stumps [1]. Penile location is rare, and differential diagnosis with severe balanoposthitis, epidermal cysts, and neoplasms can be difficult.

We report a rare case of pilonidal cyst located between coronal sulcus and prepuce which, due to its ulcerated aspect and absence of a tract with projecting hairs, simulated a penile carcinoma. 

## 2. Case Report

A 26-year-old Caucasian uncircumcised man presented with a 3-month history of an ulcerated lesion located between the coronal sulcus and the prepuce of the dorsum of his penis, not associated with pain or discharge. Past medical history disclosed that two years ago he had noticed a tiny cyst in his dorsal foreskin. On physical examination, the foreskin was swollen and a bit difficult to retract. Between the coronal sulcus and the prepuce of the dorsal aspect of the penis there was a 1.5 cm whitish ulcer overlying a hard nodule apparently fixed to the corpora cavernosa mimicking a penile carcinoma (Figure 1). Inguinal lymph nodes were not enlarged. 

The patient was admitted for surgical excision. The ulcerated lesion with the underlying nodule, hardly attached but not infiltrating Buck's fascia, was widely excised. Frozen sections of the resected specimen ruled out malignancy. Formal circumcision was performed. 

Histology showed a thick-wall cyst lined by a stratified squamous epithelium. Few hair fragments were recognizable in the lumen and in the wall itself, where they elicited a foreign body granulomatous reaction.

Recovery was uneventful, and he remains asymptomatic 10 months after surgery.

## 3. Discussion

Pilonidal sinus of the penis is a rare condition with 15 cases (including the present one) having been reported in literature. Analysis of reported cases shows that it affects uncircumcised males between 21 and 59 years of age and is usually located between coronal sulcus and prepuce, mostly dorsally (60%) or ventrally (33%). It is believed that the coronal sulcus acts as a cleft where hairs may collect and are forced to penetrate into penile shaft and foreskin by the natural movement that occurs between these two surfaces [1]. Retention of follicular products results in infection and abscess formation; thus, the sinus tract usually forms to drain the abundant suppuration mimicking severe balanoposthitis [2]. In the rare cases in which there is no secondary infection, the opening onto the skin is absent, and pilonidal cyst may form [2]. 

Our patient did not present with a sinus nor with a cyst, but with an ulcerated hard lesion without discharge or evidence of hairs projecting from it, thus mimicking penile carcinoma.

Unusual lesions of the penis should be biopsied for both bacteriological and histological assessment. Surgeons should be aware that classic penile pilonidal sinus, with hairs projecting from it, can sometimes be associated with actinomycosis needing surgical excision and long-term penicillin treatment to avoid systemic complications of this disease [2]. It is also important to be aware that penile pilonidal cyst may simulate carcinoma, like in the reported case, or may undergo malignant transformation, with epidermoid carcinoma being the most frequent lesion [3]. 

## Figures and Tables

**Figure 1:**